# A 55-Word Story Narrative Medicine Workshop to Promote Reflective Practice Among Internal Medicine Residents

**DOI:** 10.15766/mep_2374-8265.11639

**Published:** 2026-09-28

**Authors:** Anna Maria Gramelspacher, Bernice Man, Brandon Bedell, Michael Blackie

**Affiliations:** 1 Assistant Professor, Department of Medicine, University of Illinois at Chicago; 2 Associate Professor, Department of Medicine, University of Illinois at Chicago; 3 Fellow, Division of Endocrinology, Diabetes & Metabolism, Department of Medicine, Beth Israel Deaconess Medical Center; 4 Associate Professor, Department of Medical Education, University of Illinois at Chicago

**Keywords:** Health Humanities, Reflection/Narrative Medicine, Writing Workshop, Internal Medicine, Humanities (Art, Literature, Music)

## Abstract

**Introduction:**

Engaging in reflective practice is a critical component of training resident physicians toward competency in clinic practice, yet opportunities to engage in it are rare. To be effective practicing clinicians, residents must be able to reflect on and learn from their clinical work.

**Methods:**

One 60-minute 55-word story reflective writing workshop was delivered to 3 groups of second- and third-year internal medicine residents as part of a mandatory curriculum.

**Results:**

Each session was led by 2 facilitators with varying levels of training in health humanities. Sixty-one residents completed the workshop, and 54 of them completed a postworkshop survey (89% response rate). All respondents enjoyed the session, and 87% found that the session improved their connection to colleagues. Eighty-two percent of respondents agreed or strongly agreed that the workshop improved their well-being. Ninety-four percent found the session valuable. Ninety-eight percent agreed or strongly agreed that the workshop offers a safe space to process emotionally distressing and/or difficult experiences related to patient care. Seventy-five percent agreed or strongly agreed that the session improved their understanding of their role within a health care team.

**Discussion:**

This workshop is an accessible way to incorporate reflective practice into a busy resident curriculum. The facilitated close reading and writing of 55-word stories offered a safe and effective way for residents to process emotionally distressing experiences, form greater connections with colleagues, deepen their understanding of their role as health care providers, and improve well-being.

## Educational Objectives

By the end of this activity, learners will be able to:

1. Apply skills in close reading to the discussion of selected 55-word stories.
2. Engage in reflective writing within a safe space to better understand their role as health care providers and process emotionally difficult experiences related to patient care.
3. Improve their perceived well-being and connection to colleagues through shared story-telling, discussion, and reflection.

## Introduction

In *Narrative Medicine: Honoring the Stories of Illness*, Rita Charon describes the vital role reflection plays in the work of doctoring by comparing it to the systolic and diastolic function of the heart. She notes examples of *systolic* work as ordering, diagnosing, interpreting, and acting, which occur alongside the *diastolic* work of reflecting, absorbing, listening, and paying attention. She writes, “The systolic and diastolic movements of the heart together constitute cardiac function, by which the heart *acts*, and dysfunction of either is catastrophic.”^1^ When thinking of Charon's metaphor in relation to graduate medical education (GME), we find that residents spend a great deal of time doing the *systolic* work of doctoring and rarely have the opportunity to engage in the equally important *diastolic* work. The workshop we describe here offers a way to create space for this diastolic work.

Significant scholarly work over the past decades has demonstrated the need and benefit of incorporating the health humanities into medical education, as evidenced by the AAMC 2020 report, *The Fundamental Role of the Arts and Humanities in Medical Education*.^2^ More specifically, the use of health humanities pedagogy, such as narrative medicine workshops, has been explored as a means to promote competency in communication,^3-5^ critical thinking and reflective capacity,^4^ and empathy,^5^ in addition to encouraging professional identity formation^6^ in the practice of medicine. Despite the growing presence of health humanities in medical schools, less than a quarter of published studies of health humanities interventions have been conducted at the GME level.^7^ Notably, it is at this level where burnout tends to increase significantly while empathy decreases, threatening the quality of health care delivery and the well-being of physicians themselves.^8,9^ This workshop is different from previously published Narrative Medicine and Reflective Practice curricula within *MedEdPORTAL*, as the workshop occurs in just 1 mandatory session and is easily adaptable to any topic residents wish to write about reflectively.

The clinical demands of residency training are significant to provide resident physicians with adequate opportunities to achieve mastery of clinical medicine. However, to be effective practicing clinicians, residents must also be able to reflect on and learn from their clinical work. There is little dedicated time within a residency curriculum for residents to engage in reflective practice, and few published studies that describe how to achieve this. Of the published studies of narrative medicine interventions in GME, many require significantly more didactic time or are done with much smaller groups of residents.^10-14^

An hour-long narrative medicine workshop, specifically one utilizing the 55-word story, offers an accessible instructional method for residents to practice close reading skills and write about their experience as health care providers. The 55-word story is a form of creative short-story writing initially presented in 1995 by editor Steve Moss in the book *The World's Shortest Stories*.^15^ This form of story writing was subsequently taken up by 2 physicians who wrote a collection of stories about their work in medicine, publishing in *JAMA* in 2000.^16,17^ Since that time, 55-word stories have appeared many times throughout the medical literature as a way for physicians to write creatively about the work of doctoring.^18-20^

Our workshop was guided by Rita Charon's conceptual framework for the use of narrative in clinical practice: to employ skills in close reading, narrative theory, phenomenological inquiry, and creativity so that learners can recognize, absorb, interpret, and be moved by the stories of illness. The pedagogical tool of close reading is the signature method of narrative medicine. The practice of close reading asks readers to examine not only the words that make up a story, but the framing of a text, the form it has taken, and the plot and narrative arc to uncover its meaning. These skills, when learned and practiced together, can improve residents’ understanding of what their patients endure in illness, and also, as Charon notes, “what they themselves undergo in the care of the sick.”^1^

## Methods

We delivered a 60-minute workshop during a mandatory curricular time for second- and third-year internal medicine residents. Our upper-level residents were divided into 3 groups, each containing 20-25 residents. Each group was on an X+Y schedule, where they completed 4 weeks of inpatient medicine (X) followed by 2 weeks of ambulatory medicine (Y). During the Y-block, residents had 3 hours of mandatory didactic time every Wednesday morning. We delivered this workshop to each group during 1 of the group's scheduled 60-minute didactic slots. No preparatory materials were required of the residents.

Our curricular team consisted of 3 faculty—a clinical faculty (Bernice Man), a clinical/humanities faculty (Anna Maria Gramelspacher), and a humanities faculty (Michael Blackie), along with a chief resident (Brandon Bedell). Each session was led by either Anna Maria Gramelspacher or Anna Maria Gramelspacher and Michael Blackie. While Anna Maria Gramelspacher and Michael Blackie had extensive experience in leading health humanities workshops, specifically narrative medicine, others (Brandon Bedell and Bernice Man) were new to these pedagogical methods. Our sessions benefitted from the presence of a faculty member with health humanities expertise to facilitate rich discussion among learners and to summarize and link ideas to broad, overarching concepts.

The setting of this workshop occurred in a seminar-style conference room where the resident participants and facilitator sat in a circle. We chose this seating arrangement instead of a lecture-style conference room so that we could set the stage for close discussion without hierarchy. Residents were asked to put away all electronic devices for the duration of the session. A few introductory slides were presented to share the agenda and goals of the session (Appendix A).

The workshop began with a brief introduction by the facilitators, including their background and their motivation for teaching the session. The agenda and goals of the session were also presented to residents. Previously published 55-word stories were then distributed to residents (see Appendix B). Residents were given a few minutes to read on their own in silence and instructed to underline, highlight, or make notes in the margins of the text where particular words, descriptions, or phrases resonated with them. To signal when they were finished reading, residents were instructed to simply turn their paper over. After the individual reading, the instructors explained that the group would now participate in close reading of the 55-word stories.

To begin the close reading exercise, residents were asked to volunteer to read aloud a 55-word story they wished to discuss. The facilitator then asked the resident to share what they noticed in the text, or what thoughts they had about it. After thanking the resident for sharing, the facilitator paraphrased what the resident shared and then asked for more textual evidence to support their interpretation. To open the discussion to the rest of the group, residents were asked, “What more can we find in this story?” As the discussion continued, the facilitator continued to thank residents for their comments, being careful not to judge the comments themselves, but rather link ideas and observations to others that have been made. This process continued as time permitted. For the 60-minute session, we spent about 30 minutes discussing approximately 5 stories. At the conclusion of the close reading, the facilitator summarized the process, noting that together we constructed a larger narrative.

Following the close reading exercise, the facilitator explained to residents that they would now have the opportunity to write their own 55-word story. Residents were instructed to use the blank side of their handout to write their own 55-word story based on an experience in medicine. Residents wrote silently for 5-7 minutes. During our sessions, facilitators also used this time to write their own story.

Once finished writing, the facilitators asked if any residents would like to volunteer to read their story aloud to the group. At this time, the facilitator took a moment to remind residents that the session was confidential and to review the Amherst Writing Principles in the session lesson plan (Appendix B). The first volunteer was asked to read their story aloud while the group listened, pausing after reading so that the group could reflect. The resident was asked to refrain from commenting on their own writing, and instead the facilitator began another close reading exercise of the story that had just been read aloud. This process continued until there were no more volunteers, or until the designated time in the lesson plan had elapsed. If desired, the facilitators could also share their stories, but our sessions had so many resident volunteers that we chose not to do this.

The session concluded with ample time for residents to complete a session evaluation. Residents were asked to evaluate the workshop by completing an anonymous postworkshop questionnaire. We provided 5 minutes at the end of the session to maximize response rate. A QR code was presented on the slides for residents to scan. The QR code was linked to a brief anonymous questionnaire (Appendix C) in REDCap. We designed the questionnaire to be brief and applicable to future workshops so that we could be able to compare assessments across different topics. The questionnaire used a 5‑point Likert agreement scale ranging from 1 (strongly disagree) to 5 (strongly agree) across 6 statements.

Each workshop included 1 designated curricular team member who took notes throughout the session. This team member would occasionally participate in the discussion, but their primary focus was to take notes on timing of the session, participant comments, and general observations. This was particularly helpful when reviewing the session afterward and making small changes for future sessions. For example, in our first workshop, when residents shared their stories, they would read the story and then offer a lengthy explanation, which took up considerable time and detracted from the close reading of the 55-word story itself. For our subsequent sessions, we made clear that residents were to read just the 55-word story, and then we proceeded with a close reading discussion of the just-written story. This change allowed time for more residents to share and to practice the skills in close reading. Guiding our discussion in this way encouraged the residents to absorb, interpret, and be moved by the stories of their colleagues.

Our curricular team set aside a designated time to debrief following each workshop, in which we reviewed the results of the surveys and the session notes, as well as our own reflections about the teaching of the session. These meetings reinforced our own reflective practice as educators. Not only did our discussions support changes to our instructional methods, but it was also helpful for our new facilitators to gain insight and confidence in how they might facilitate on their own in the future.

## Results

A total of 61 residents participated in the workshop, and 54 of them completed the brief questionnaire (89% response rate). The majority of participants were categorical internal medicine residents, whereas a few in each group were medicine/pediatrics residents.

All respondents agreed (24%) or strongly agreed (76%) that they enjoyed the 55-word workshop, and 94% of residents agreed or strongly agreed that they found the session to be valuable. Ninety-eight percent of residents agreed (35%) or strongly agreed (63%) that the workshop offered a safe space to process emotionally distressing and/or difficult experiences related to patient care. A total of 87% of residents agreed or strongly agreed that the session improved their connection to colleagues. Although 4% of residents disagreed or strongly disagreed that the session improved their understanding of their role as part of a health care team, 75% agreed or strongly agreed. Eighty-two percent of residents agreed or strongly agreed that the 55-word workshop improved their well-being. The results are summarized in the Figure.

**Figure.**
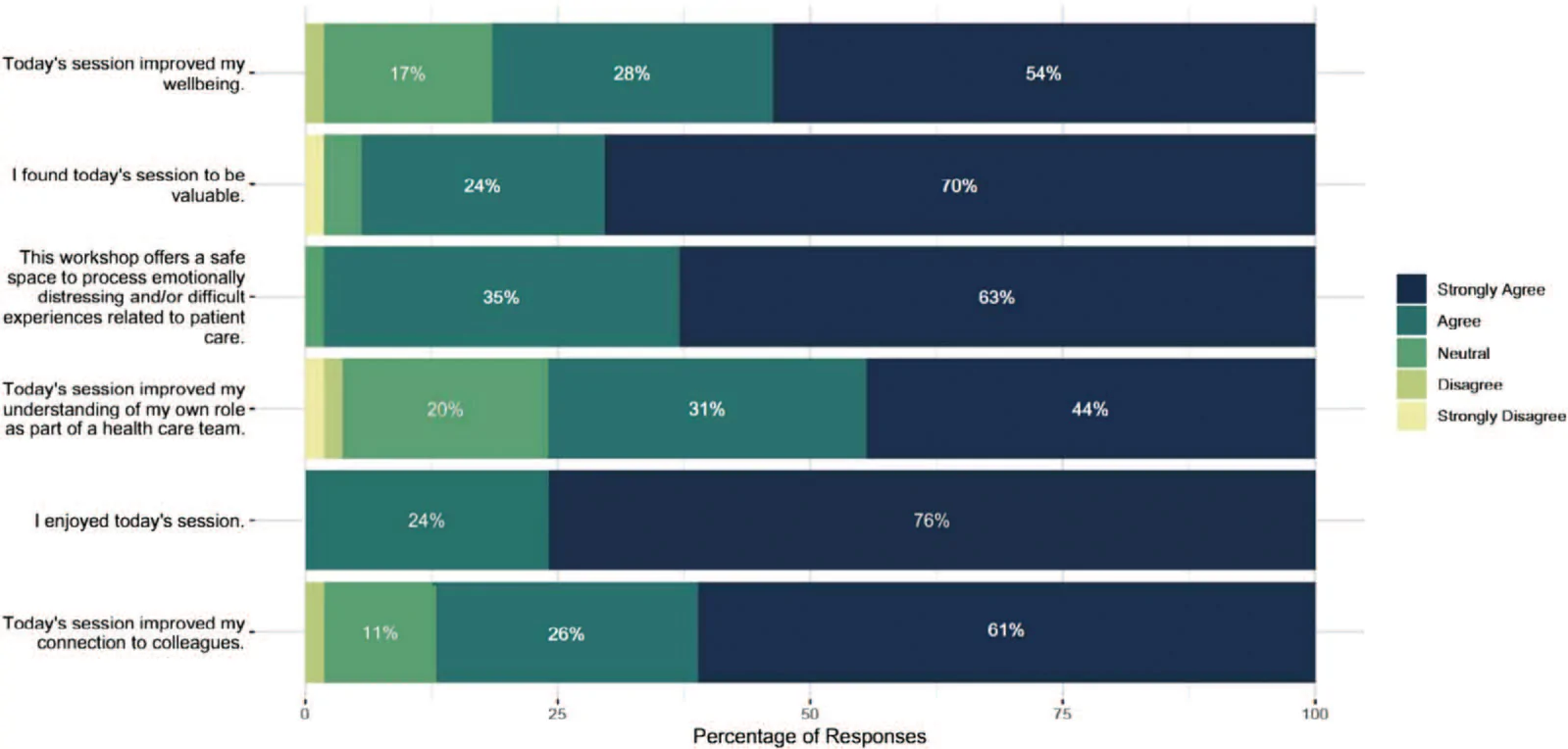
Distribution of participant responses (*N* = 54) to the 55-word writing workshop evaluation statements using a 5-point Likert scale (1 = *strongly disagree*, 5 = *strongly agree*).

We did not collect stories for analysis at the end of the workshop, but our session notes detailed common themes that arose among the 55-word stories. Many residents shared stories of losing a patient and of personal connections to patients. Some shared patient stories that caused them moral distress, whereas a few shared humorous accounts of their days as residents.

## Discussion

This workshop offered a way to incorporate reflective practice into a busy resident curriculum requiring minimal resources, low time commitment (60 minutes), and no preparation for residents. The results from residents were overwhelmingly positive. Residents found the workshops valuable and that participation in the workshop improved their well-being and connection to colleagues.

Most significantly, the facilitated close reading and writing of 55-word stories created a safe space for residents to better understand their role as health care providers and process emotionally difficult experiences related to patient care. What we heard during the sharing of the 55-word stories are the stories residents carry. The stories shared by residents were often ones of losing a patient, making a connection with a patient or family, or of their own limitations. This workshop provided an opportunity to vocalize, share, and learn from their experiences. Reflective practice may intimidate learners, especially when it requires that they write thoughtfully about their professional experiences. A counterintuitive aspect of these stories is that their restrictive word count makes the exercise conceptually manageable in ways that open-ended writing exercises do not. Distilling an experience from one's practice down to 55 words is freeing, even liberating.

As facilitators, we wrote our own stories alongside residents. In doing so, we reinforced that reflective practice is important for new and seasoned clinicians alike; we are writing and learning and reflecting alongside them. By utilizing a structured close reading framework in the discussion of the stories and written work, our sessions took on a rhythmic cadence that soon became second nature to facilitators and participants. This structured pedagogical framework, similar to that imposed by the 55-word story writing exercise, created a safe space for residents and facilitators to reflect, learn, and further develop their skills. While not every resident completed a finished 55-word story or chose to share their written work, resident responses to participating in the workshop were overwhelmingly positive. While the 55-word stories that were shared offered powerful reflections of their work as residents, the dedicated space to reflect and share may have a lasting effect on residents’ well-being, their ability to process emotionally difficult experiences related to patient care, their understanding of their role as a member of a health care team, and their connection to colleagues.

As stated previously, the implementation of our workshop required trained and willing facilitators to lead and guide discussion for residents. While many practicing clinicians can learn to facilitate, the quality of the workshop and subsequent discussion is enhanced by experienced educators comfortable with small-group facilitation and expertise in close reading. Our workshops benefitted from partnering clinician educators with experienced humanities faculty, and we encourage others to pursue this option when possible. This workshop offers an excellent opportunity to form cross-disciplinary partnerships with humanities experts. Regardless of the level of facilitator expertise, we encourage anyone delivering this workshop to dedicate 1 co-facilitator as an observer and note-taker during the session. Furthermore, we recommend that facilitators set aside time together following the workshop to review session feedback and reflect.

While our workshops were conducted with senior internal medicine residents, this workshop can be generalized to many specialties within GME. Previously published studies have demonstrated effective narrative medicine interventions in OB/GYN, emergency medicine, and pediatrics residency programs, to name a few.^6,7,9^ This workshop could be further generalized to medical student learners during clerkship rotations, or even in faculty development sessions. While we selected 2 specific 55-word story compilations that portrayed a broad range of topics related to medicine,^19,20^ there are many other previously published 55-word stories specific to a wide range of specialties such as military medicine,^21^ family medicine,^16,22^ and psychiatry^16,20^ and to topics ranging from COVID-19^23,24^ to reflections on being medical student.^25^Additional stories can be found on many online forums such as the American Medical Association blog^25^ and medical school blogs.^23,24^

Regardless of specialty or level of training, reflective practice is a lifelong skill that benefits learners and practicing clinicians across specialties and levels. Our workshops occurred with just 61 internal medicine residents at 1 academic teaching hospital. Further investigation of the implementation and outcomes of this workshop in other settings would strengthen the data and findings.

A challenge for other institutions may be getting administrative support or buy-in. The year prior to implementing this workshop, we piloted a few humanities sessions during which we gathered feedback from residents and presented it to the curricular committee of our program. The pilot data was pivotal in getting this mandatory curricular workshop approved, as well as our ability to link it to developing ACGME competencies such as Practice-Based Learning and Improvement, Interpersonal and Communication Skills, and Professionalism.

In conclusion, our workshop offered an effective and enjoyable way for residents to participate in the vital *diastolic* work of doctoring—a lifelong practice that may lead to more fulfilling work, greater connection to colleagues, and more holistic and empathic care of patients and themselves as care providers.

## Appendices


55-Word Story Workshop.pptxLesson Plan.docxWorkshop Survey.docx
*All appendices are peer reviewed as integral parts of the Original Publication.*
