# Supplementary material for: A 55-Word Story Narrative Medicine Workshop to Promote Reflective Practice Among Internal Medicine Residents
Source: MedEdPORTAL. 2026 Sep 28;22:11639. doi: 10.15766/mep_2374-8265.11639 (PMC13617140; doi:10.15766/mep_2374-8265.11639)
Supplement: Supplementary file 1 [file mep_2374-8265.11639-s001.zip › B. Lesson Plan.docx]

**55-Word Story Workshop Lesson Plan**

Session Objectives:

1) Apply skills in close reading to the discussion of selected 55-word stories
2) Engage in reflective writing in order to better understand their role as health care providers and to process emotionally difficult experiences related to patient care
3) Improve their wellbeing and connection to colleagues through shared story-telling, discussion and reflection

Supplies Needed:

- Printed Copies of previously published 55-word stories^1,2^ (We like to print each publication on separately so that participants have a blank side to write on for the reflective writing portion)
- Writing Utensil for each participant
- Powerpoint (optional)
- Surveys (optional)

Lesson Plan:

| 0 – 5 min | Introductions & Review Session Objectives:   - Introduce yourself to participants if they do now already know you, also share your motivation for presenting this workshop and what you hope they will take away - You can also make some connections to clinical practice to help get participant “buy-in” (improving skills in close reading leads to improved skills in close listening leads to better diagnosis, improved doctor/patient relationships, etc) |
| --- | --- |
| 5 – 10 min | Distribute printed copies of stories to participants (*Years of Stories*^1^*, Stories in Training*^2^*)*  Residents read stories quietly, independently   - Instruct them to be interactive with the text – underline, circle, write in the margins, etc. |
| 10 – 30 min | Discussion of stories:   - Ask for a volunteer to read aloud a story they want to discuss - Discuss individual story: What happened? What was interesting to you about it? What resonated with you? Encourage participants to find examples within the text. If they draw a conclusion, ask them to explain how they got there (what in the story makes you say X?) - This part of the session can go as long as time allows (if you have 90 minutes, feel free to extend this as able) |
| 30 – 40 min | Residents write their own stories  Instructors also write their own stories  You may preface this by saying that participants will be invited to share in the end, but no one has to share their story. The purpose is to reflect and write. Their finished story does not need to be perfect or complete. The focus here is reflection. |
| 40 – 50 min | Invite participants to share their stories aloud. If a participant chooses to read the 55-word story, ask them to pause after reading so that others have a chance to respond to the written work. Participants can also share what the process was like. Let the residents lead.  It may be helpful to review Amherst Writers & Artists Essential Practices:  The following practices establish a safe environment where everyone is free to explore within their own writing and listen to each other with respect.   - Everyone’s writing, including the leader’s, is treated with equal respect and value. - Writing is kept confidential and treated as fiction. - Writers can refrain from reading their work aloud. - Responses to just-written work reflect what is strong and successful. - Responses and exercises support the development of literary craft. - When Listening in an AWA workshop we enter the universe that the writer has created and leave our assumptions behind. We are asked to leave behind our own expectations and experiences. In an AWA workshop we listen for and notice what works. We listen for and notice the choices a writer has made that help to create success in the writing. We listen without preconceived ideas about what the story should be about, how the poem should sound, or what we might do differently. |
| 50 – 55 min | Concluding remarks (see article: *Commentary on Stories in Training^3^*): Thank participants for their presence and contributions to the session. You may offer an overall reflection on the session, how the tools of close reading can inform the clinical encounter, etc. Summarize key themes and important topics that were learned/discussed/discovered. |
| 55 – 60 min | Survey Completion |

References:

1. Christianson AL. Years of stories. *JAMA*. 2009;302(10):1042. https://doi.org/10.1001/jama.2009.1203
2. Goldenberg B, Srisuwananukorn A, Cummins JS, Kreston R, Gramelspacher AM, King R. Stories in training. *Acad Med*. 2020;95(10):1540-1540. doi:10.1097/ACM.0000000000003319
3. Gramelspacher AM, Cummins JS. Stories in training commentary on stories in training. *Acad Med*. 2020;95(10):1541. doi:10.1097/ACM.0000000000003319
