# Supplementary material for: A 55-Word Story Narrative Medicine Workshop to Promote Reflective Practice Among Internal Medicine Residents
Source: MedEdPORTAL. 2026 Sep 28;22:11639. doi: 10.15766/mep_2374-8265.11639 (PMC13617140; doi:10.15766/mep_2374-8265.11639)
Supplement: Supplementary file 1 [file mep_2374-8265.11639-s001.zip › C. Workshop Survey.docx]

# Confidential

*Page 1*

Lecture date

I enjoyed today's session.	Strongly Agree

Agree Neutral Disagree

Strongly Disagree

I found today's session to be valuable.	Strongly Agree Agree

Neutral Disagree

Strongly Disagree

Today's session improved my wellbeing.	Strongly Agree Agree

Neutral Disagree

Strongly Disagree

Today's session improved my connection to colleagues.	Strongly Agree

Agree Neutral Disagree

Strongly Disagree

This workshop offers a safe space to process	Strongly Agree

emotionally distressing and/or difficult experiences	Agree

related to patient care.	Neutral

Disagree

Strongly Disagree

Today's session improved my understanding of my own	Strongly Agree role as part of a health care team	Agree

Neutral Disagree

Strongly Disagree

Would you like to provide additional comments or	Yes

feedback for this session, or humanities curricular	No content in general?

Please provide any other thoughts/ feedback/suggestions.**!Unexpected End of Formula**
